# Novel scheme for secure data transmission based on mesoscopic twin beams and photon-number-resolving detectors

**DOI:** 10.1038/s41598-022-19503-y

**Published:** 2022-09-16

**Authors:** Alessia Allevi, Maria Bondani

**Affiliations:** 1 Department of Science and High Technology, University of Insubria, Via Valleggio 11, 22100 Como, Italy; 2 Institute for Photonics and Nanotechnologies, IFN-CNR, Via Valleggio 11, 22100 Como, Italy

**Keywords:** Quantum information, Quantum optics

## Abstract

Quantum resources can improve the quality and security of data transmission. A novel communication protocol based on the use of mesoscopic twin-beam (TWB) states of light is proposed and discussed. The message sent by Alice to Bob is encoded in binary single-mode thermal states having two possible mean values, both smaller than the mean value of the TWB. Such thermal states are alternately superimposed to the portion of TWB sent to Bob. We demonstrate that in the presence of an eavesdropping attack that intercepts and substitutes part of the signal with a thermal noise, Bob can still successfully decrypt the message by evaluating the noise reduction factor for detected photons. The protocol opens new perspectives in the exploitation of quantum states of light for applications to Quantum Communication.

## Introduction

Quantum resources have the potential to improve the secure transmission of information between two parties. Till now, Quantum Communication protocols have been implemented^1–4^ by means of entangled states produced at the single-photon level^5–8^. In this case the information to be sent is encoded in the different degrees of freedom of the quantum states, such as polarization, transverse momentum, time-bin, and orbital angular momentum^9–12^. The success of the protocols relies on the high rate at which the information is transmitted, which compensates for possible eavesdropper’s attacks^13^.

At variance with the single photon domain, where the presence of losses can reduce the rate at which the information is sent, in the mesoscopic one the optical pulses contain sizeable numbers of photons, thus resulting more robust against any kind of external degradation^14,15^. In a recent work of ours we have demonstrated that the transmission of one of the parties of a twin-beam (TWB) state through a lossy and noisy channel does not prevent the observation of nonclassical correlations between signal and idler arms^16^. To this aim, we have considered the nonclassicality criterion based on the noise reduction factor^17^ and proved that it can be written in terms of measurable quantities also including the drawbacks that can affect the communication, namely loss and noise sources. This means that from the experimental measurement of the noise reduction factor it is possible to extract information not only about the quantum states, but also on the drawbacks.

Based on these considerations, in the following we propose a new protocol to transmit information between two parties apart from each other: Alice generates a mesoscopic TWB state and sends one portion to Bob. Superimposed to Bob’s portion she also sends the information encoded in sequences of binary signals given by two single-mode thermal states differing from each other for the mean value. Both Alice and Bob measure their portions, and then Alice sends the results of her measurements to Bob. By the measurement of the photon-number correlations, Bob can decode the message. If in the meanwhile an eavesdropper’s attack has occurred, the comparison between the correlation measurements and the theoretical model can reveal it and thus the sequence under attack can be discarded. This protocol opens new perspectives in the field of Quantum Communication showing that also mesoscopic quantum states can find useful applications. The work is organized as follows: in “Results” section the different steps of the protocols are presented. In the same section we consider a realistic situation to explore the role of the length of the data sequence for the successful implementation of the protocol. “Discussion” section is devoted to the possible eavesdropper’s attacks and to the strategy used to reveal them. Finally, in the “Conclusions” we summarize our results and outline some future perspectives.

## Results

### The protocol step-by-step

As already anticipated in the “Introduction”, Alice produces mesoscopic TWB states. We assume that such states are described as the tensor product of $\mu$ identical (*i*.*e*. equally populated) TWB states

1 $$\begin{aligned} |\psi _{\mathrm{TWB}} \rangle = \sqrt{1-\lambda ^2} \sum _{n_k = 0}^{\infty } \lambda ^{n_k} |n_k,n_k \rangle , \end{aligned}$$

where $\lambda ^2 =\langle n_k \rangle /(1+\langle n_k \rangle )$ and $\langle n_k \rangle$ is the mean number of photons in the *k*-th mode^17,18^.

The multi-mode TWB is described by the following density matrix

2 $$\begin{aligned} \rho _{\mathrm{TWB,~\mu }} = \sum _{n=0}^{\infty } P^{\mu }(n) |n, n\rangle \langle n, n|, \end{aligned}$$

where $|n\rangle =\delta (n- \sum _{k=1}^{\mu }n_k) \bigotimes _{k=1}^{\mu } |n_k\rangle _k$ and *n* is the overall number of photons in the $\mu$ spatio-spectral modes that impinge on the detector, while $P^{\mu }(n)$ is the multi-mode thermal distribution

3 $$\begin{aligned} P^{\mu }(n) = \frac{(n+ \mu -1)!}{n! (\mu -1)!\left( \langle n \rangle /\mu +1\right) ^{\mu }\left( \mu /\langle n \rangle +1\right) ^n}, \end{aligned}$$

in which $\langle n \rangle$ is the mean number of photons in each arm. These states are entangled in the number of photons. To prove it, many nonclassicality criteria have been proposed during the past twenty years^19–22^. Some of them are necessary and sufficient, some are only sufficient, and some others only necessary^23^. In this work, we consider a nonclassicality criterion based on the noise reduction factor^24^, since it can be easily accessed from the experimental point of view^25^. We define the noise reduction factor as^26^

4 $$\begin{aligned} R = \frac{\sigma ^2(n_1-n_2)}{\langle n_1 \rangle + \langle n_2 \rangle }, \end{aligned}$$

where $\sigma ^{2}(n_1 - n_2)$ is the variance of the distribution of the photon-number difference between the two parties, while $\langle n_1 \rangle + \langle n_2 \rangle$ is the shot-noise-level, that is the variance of the distribution of the photon-number difference in the case of two coherent states having mean values $\langle n_1 \rangle$ and $\langle n_2 \rangle$. It can be demonstrated that the condition $R<1$, meaning that signal and idler arms exhibit sub-shot-noise correlations, represents a sufficient condition for entanglement^23,24^. We have already shown that *R* can be also written in terms of measurable quantities, such as detected photons^17^, so that the nonclassicality condition can be directly applied to the experimental data

5 $$\begin{aligned} R &= \frac{\sigma ^2(m_1 - m_2)}{\langle m_1 \rangle + \langle m_2 \rangle }= \frac{\langle (m_1 - m_2)^2\rangle - \langle (m_1 - m_2)\rangle ^2}{\langle m_1 \rangle + \langle m_2 \rangle } \\ & = \frac{\langle m_1^2\rangle + \langle m_2 ^2\rangle - 2\langle m_1 m_2 \rangle - (\langle m_1 \rangle ^2 + \langle m_2 \rangle ^2 - 2 \langle m_1 \rangle \langle m_2 \rangle )}{\langle m_1 \rangle + \langle m_2 \rangle } . \end{aligned}$$

In particular, for a multi-mode TWB state, $\langle m_1 m_2 \rangle = (1+1/\mu ) \langle m_1 \rangle \langle m_2 \rangle + \sqrt{\eta _1 \eta _2} \sqrt{\langle m_1 \rangle \langle m_2 \rangle }$, so that the noise reduction factor can be expressed as

6 $$\begin{aligned} R = 1- \frac{2\sqrt{\eta _1 \eta _2}\sqrt{\langle m_1 \rangle \langle m_2 \rangle }}{\langle m_1 \rangle + \langle m_2 \rangle } + \frac{(\langle m_1 \rangle - \langle m_2 \rangle )^2}{\mu (\langle m_1 \rangle + \langle m_2 \rangle )}, \end{aligned}$$

$\eta _j$ being the quantum efficiencies of the detectors and $\langle m_j \rangle = \eta _j \langle n_j \rangle$ the mean values of detected photons. The equation can be further simplified by assuming $\langle m_1 \rangle = \langle m \rangle = \eta \langle n \rangle$, $\langle m_2 \rangle = t \langle m \rangle = t \eta \langle n \rangle$, $t \in [0,1]$ being the transmission efficiency quantifying the balancing level

7 $$\begin{aligned} R = 1- \frac{2\eta t \langle m \rangle }{(1+t) \langle m \rangle } + \frac{(1-t)^2 \langle m \rangle ^2}{\mu \left[ (1+t) \langle m \rangle \right] }. \end{aligned}$$

Now, let us assume that Alice encodes a binary message in the portion of multi-mode TWB sent to Bob by adding a small noise signal. This is given by a single-mode thermal state with two possible mean values: the higher mean value $\langle m_{H} \rangle$ corresponds to the logic bit 1, while the lower mean value $\langle m_{ L} \rangle$ to the logic bit 0. We can assume that $\langle m_{H} \rangle$ is 3 or 4 times $\langle m_{L} \rangle$, and that they represent the 20$\%$ or the 7$\%$ of the global light, respectively. As better remarked in the following, we choose to encode the message in a single-mode thermal state because the expression of *R* is particularly sensitive to the presence of added thermal noise^16^. The presence of this noise source modifies the statistics of light, which becomes the convolution between the multi-mode thermal distribution of the TWB and the single-mode thermal state with $\mu _{\mathrm{TH}} = 1$ of the additional noise

8 $$\begin{aligned} P^{\mu ,\mu _{\mathrm{TH}}}(n) = \sum _{n=0}^m P^{\mu _{\mathrm{TH}}}(m-n) P^{\mu }(n). \end{aligned}$$

**Figure 1:**
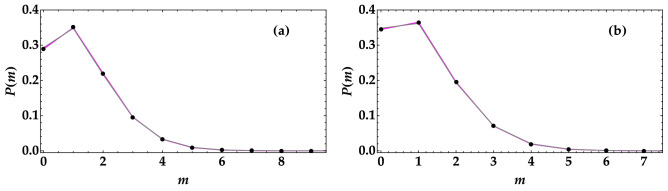
Experimental reconstruction of the distribution of detected photons when a single-mode thermal state is superimposed to a portion of TWB. Black dots: data; magenta curve: fitting function according to Eq. (3); gray curve: fitting function according to Eq. (8). In the left panel the mean value of the state is $\langle m \rangle = 1.27$, whereas in the right panel is $\langle m \rangle = 1.07$. In both cases the fidelity of data to the two theoretical descriptions is very high ($f>0.9999$), thus proving that the presence of a small thermal noise signal cannot be simply discriminated by analyzing the statistics.

To investigate the impact of an added thermal noise on the detected-photon statistics, in Fig. 1 we show data taken for two different mean values of the thermal noise (black dots) together with the theoretical fitting functions according to a multi-mode thermal distribution (Eq. (3), magenta curve) and the convolution of it with a single-mode thermal statistics (Eq. (8), gray curve). From the plots it can be observed that the two curves are both well superimposed to data, as proved by the high values of the fidelity $f = \sum _{m=0}^{\bar{m}} \sqrt{P(m) P_{\mathrm{theo}}(m)}$, where *P*(*m*) and $P_{\mathrm{theo}}(m)$ are the experimental and theoretical distributions, respectively, and the sum is extended up to the maximum detected-photon number, $\bar{m}$, above which the two distributions become negligible. This demonstrates that it is really hard to discriminate the presence or not of an additional thermal noise. Nevertheless, by comparing the statistics shown in the two panels, we notice that having access to the statistics of light could be sufficient to discriminate which signal was sent. However, this operation requires a proper data sample. If the sequence is too short, the reconstructed statistics is not reliable. Moreover, in the case of an eavesdropping attack, in which part of the sent signal is intercepted and additional noise is added, the reconstruction of the statistics can no longer be used to discriminate which light signal has been sent. On the contrary, evaluating the level of nonclassicality can help since both loss and noise sources can be incorporated in the model for the noise reduction factor and extracted from the fit of the measured value of *R*. In particular, in the case of an ideal transmission channel, in which the signal is encoded in a single-mode thermal state with $\mu _{\mathrm{TH}} =1$ superimposed to the portion of TWB, the expression in Eq. (7) modifies as

9 $$\begin{aligned} R& = 1- \frac{2\eta t \langle m \rangle }{(1+t) \langle m \rangle + \langle m_{\mathrm{TH}} \rangle } + \frac{(1-t)^2 \langle m \rangle ^2}{\mu \left[ (1+t) \langle m \rangle + \langle m_{\mathrm{TH}} \rangle \right] } \\ & \quad + \frac{\langle m_{\mathrm{TH}} \rangle ^2}{\left[ (1+t)\langle m \rangle + \langle m_{\mathrm{TH}} \rangle \right] }, \end{aligned}$$

where *t* quantifies the balancing between signal and idler arms. According to this expression, the condition $R<1$ is satisfied only if the mean value of the single-mode thermal noise is properly limited, namely

10 $$\begin{aligned} \langle m_{\mathrm{TH}} \rangle < \sqrt{\left[ 2 \eta t \mu - (1-t)^2 \langle m \rangle \right] \frac{\langle m \rangle }{\mu }}. \end{aligned}$$

In the communication protocol, the two mean values of the thermal noise are chosen so that in both cases the nonclassicality condition is satisfied. The value of the bit of information can be extracted from the noise reduction factor, which is much more sensitive than the statistics, as better shown in the next Sections.

In the case of an eavesdropper’s attack, the expression of *R* is modified. Indeed, in order to intercept the message, Eve can pick up part of the signal transmitted to Bob and thus evaluate the nonclassicality level as

11 $$\begin{aligned} R_{\mathrm{\mathbf{E}}} = 1- \frac{2\eta t \langle m \rangle }{(1+t) \langle m \rangle +t \langle m_{\mathrm{TH}} \rangle } + \frac{(1-t)^2 \langle m \rangle ^2}{\mu \left[ (1+t) \langle m \rangle + t \langle m_{\mathrm{TH}} \rangle \right] } + \frac{t^2 \langle m_{\mathrm{TH}} \rangle ^2}{\left[ (1+t)\langle m \rangle + t \langle m_{\mathrm{TH}} \rangle \right] }. \end{aligned}$$

In the meanwhile, Bob will measure

12 $$\begin{aligned} R_{\mathrm{\mathbf{B}}} = 1- \frac{2\eta (1-t) \langle m \rangle }{(2-t) \langle m \rangle +(1-t) \langle m_{\mathrm{TH}} \rangle } + \frac{(t)^2 \langle m \rangle ^2}{\mu \left[ (2-t) \langle m \rangle + (1-t) \langle m_{\mathrm{TH}} \rangle \right] } + \frac{(1-t)^2 \langle m_{\mathrm{TH}} \rangle ^2}{\left[ (2-t)\langle m \rangle + (1-t) \langle m_{\mathrm{TH}} \rangle \right] }. \end{aligned}$$

To cover the effect of the attack, Eve superimposes to the light signal sent to Bob a thermal noise having the same mean value of the subtracted light, namely $t (\langle m \rangle + \langle m_{\mathrm{TH}} \rangle )$. Thus, Bob measures

13 $$\begin{aligned} R_{\mathrm{\mathbf{B,TH}}} & = 1- \frac{2\eta (1-t) \langle m \rangle -(1-t)^2 \langle m_{\mathrm{TH}} \rangle ^2-t^2 (\langle m \rangle + \langle m_\mathrm{TH}\rangle )^2}{(2-t) \langle m \rangle +(1-t) \langle m_{\mathrm{TH}} \rangle +t \langle m \rangle +t \langle m_{\mathrm{TH}} \rangle }\nonumber \\ & \quad + \frac{(t)^2 \langle m \rangle ^2}{\mu \left[ (2-t) \langle m \rangle + (1-t) \langle m_{\mathrm{TH}} \rangle +t \langle m \rangle + t \langle m_{\mathrm{TH}} \rangle \right] }. \end{aligned}$$

Note that the successful implementation of the protocol relies on the value of *t*, which quantifies the amount of signal subtracted to Bob. If this value is small enough, Bob can still reveal sub-shot-noise correlations and also estimate the binary message encoded in the thermal noise by Alice, while Eve is not able to obtain enough information from the data, neither by evaluating the statistics nor by comparing the noise reduction factor. In addition, measured values of *R* larger than 1 must be interpreted as the result of an Eve’s attack, thus suggesting the interruption of the communication. We also notice that Bob can use a portion of the dataset to extract the value of *t* by performing a fitting procedure according to Eq. (13). This allows him to understand if an eavesdropper’s attack has occurred.

### A realistic example

As described in the previous Section, to perform the protocol, Alice sends to Bob the message encrypted in the thermal noise superimposed to Bob’s TWB part, repeated in short sequences. The length of the sequences represents an important parameter to control. In fact, if the sequence is long enough, the message can be decoded by simply recontructing the statistics of light, so that the strategy would not be safe since an eavesdropper could intercept the communication and also modify it.

**Figure 2:**
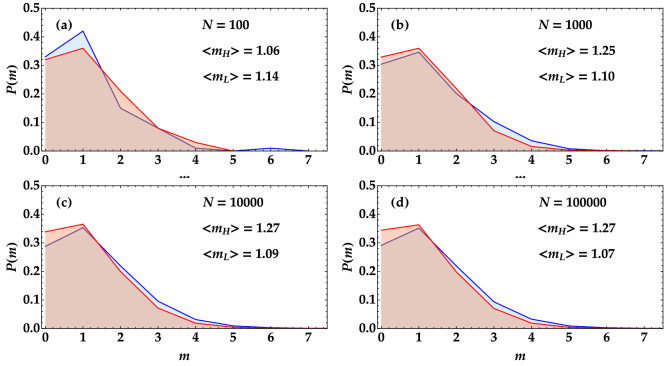
Experimental reconstruction of the distribution of detected photons when a single-mode thermal state is superimposed to a portion of TWB. The blue curves correspond to the state with the higher mean value, whereas the red curve to the lower mean value. The different panels correspond to different number of shots used to reconstruct such statistics.

To better emphasize this point, in the four panels of Fig. 2 we show the photon-number distributions of the light states given by a portion of TWB superimposed to a single-mode thermal state. The red surface corresponds to the thermal state with higher mean value, while the blue surface to the thermal state with lower mean value. In panel (a) the reconstruction has been obtained from 100 shots, in panel (b) from 1000 shots, in panel (c) from 10,000 shots, and in panel (d) from 100,000 shots. As it can be easily noticed, the mean value of the distributions changes according to the number of shots. In particular, in case (a) its evaluation is completely wrong since $\langle m_{H} \rangle < \langle m_{L} \rangle$, and the reconstructed distribution of detected photons is not correct.

**Figure 3:**
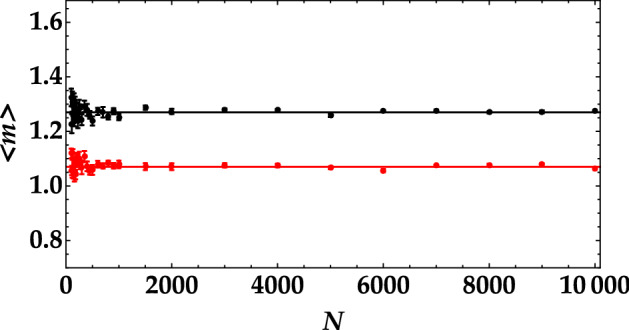
Mean number of detected photons as a function of the number of shots in the case of the higher mean value (black dots) and of the lower mean value (red dots).

**Figure 4:**
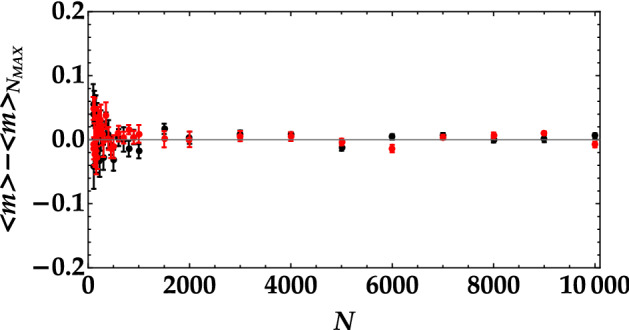
Difference between the mean number of detected photons calculated over *N* shots and that corresponding to 100,000 shots as a function of the number of shots in the case of the higher mean value (black dots) and of the lower mean value (red dots).

To better investigate the minimum number of data needed to properly discriminate the states, in Fig. 3 we show their mean values as a function of the number of shots. It clearly emerges that the determination of the mean value is noisy only for a number of pulses less than 1000, while, for larger values, the discrimination between the two thermal-noise values is easy. The same conclusion can be obtained by evaluating the difference between the mean value of the state calculated over *N* shots and that corresponding to 100,000 shots. In Fig. 4 the data corresponding to the higher mean value are shown in black, and those corresponding to the lower mean value are in red. Also this representation proves that sequences shorter than 1000 pulses do not allow for a perfect discrimination of the states by simply calculating the mean value. Moreover, it proves that this is valid for both choices of additional thermal noise.

To decript the message encoded in the two single-mode thermal states, Bob measures the shot-by-shot number of photons and evaluates the noise reduction factor using Alice’s measurements of her part of the TWB, which he must receive separately. Indeed, the calculation of the noise reduction factor makes it possible to distinguish between the two signals even if an eavesdropper intercepts the message and introduces a noise source with the same mean value as the subtracted signal. On the contrary, in that case the mean value is useless to decrypt the message.

**Figure 5:**
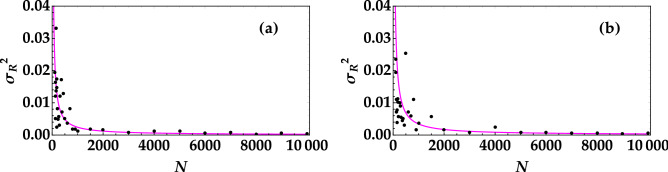
Variance of the noise reduction factor as a function of the number of shots in the case of the higher mean value (left panel) and of the lower mean value (right panel). In each plot the magenta curve is the theoretical fitting function $y=a/x$, with $a = 2.4 \pm 0.3$ in (**a**) and $a = 3.4 \pm 0.8$ in (**b**), which is valid in the case of randomly distributed errors.

**Figure 6:**
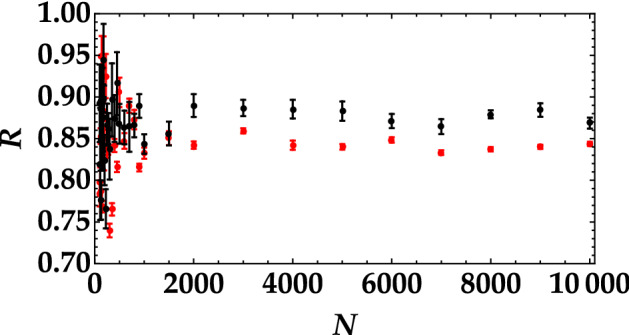
Noise reduction factor as a function of the number of shots in the case of the higher mean value (black dots) and of the lower mean value (red dots).

Also in the case or *R*, it is crucial to investigate which is the minimum number of shots necessary to properly discriminate the two signals. To this aim, in Fig. 5 we plot the variance of *R* as a function of the number of pulses, *N*. The data are shown as dots, while the fitting function $y=a/x$, *a* being a positive constant, is shown as magenta curve. The two panels correspond to the different mean values of the employed optical states. The two curves allow us to set a threshold on the minimum number of shots necessary to decrypt the message. The same result can be achieved by considering the noise reduction factor for the two states as a function of the number of shots (see Fig. 6). We can clearly see that for values of *N* smaller than 1000 the error in the determination of *R* is comparable to the difference between the mean values of the two thermal noise signals. These results prove that, in the absence of attacks, using either the mean value or the noise reduction factor is a good strategy to discriminate which signal has been sent.

## Discussion

According to the model presented in the previous Section, here we consider the case in which the sequence of data is intercepted by Eve. In this situation, part of the signal is subtracted by the eavesdropper, thus introducing a loss, and at the same time a new signal is inserted in the communication channel. This new signal, whose mean value is equal to that of the subtracted amount, can be either a thermal signal or a coherent one.

For instance, let us assume that it is a single-mode thermal state. We consider a TWB state having mean value equal to $\langle m \rangle = 2$ and $\mu = 100$ and two single-mode thermal states encoding the message with mean values $\langle m_{H} \rangle = 0.3$ and $\langle m_{H} \rangle = 0.1$, respectively.

**Figure 7:**
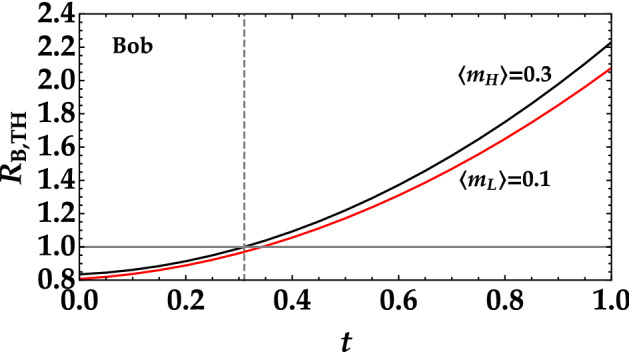
Noise reduction factor measured by Bob as a function of the transmittance efficiency *t* in the case of the higher mean value (black curve) and of the lower mean value (red curve). The quantum efficiency $\eta$ is set equal to 0.2, which is the typical value obtained with HPDs. The horizontal gray line at $R=1$ represents the boundary between classical and nonclassical correlations. The vertical line corresponds to $t=t^*$, that is the threshold value over which Bob measures $R>1$.

**Figure 8:**
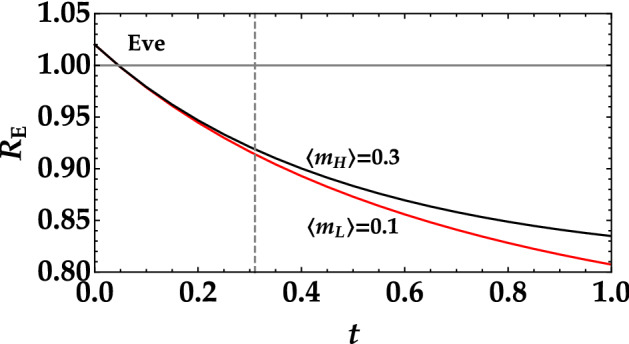
Noise reduction factor measured by Eve as a function of the transmittance efficiency *t* in the case of the higher mean value (black curve) and of the lower mean value (red curve). The quantum efficiency $\eta$ is set equal to 0.2. The horizontal gray line at $R=1$ represents the boundary between classical and nonclassical correlations. The vertical line corresponds to $t=t^*$, that is the threshold value over which Bob measures $R>1$.

**Figure 9:**
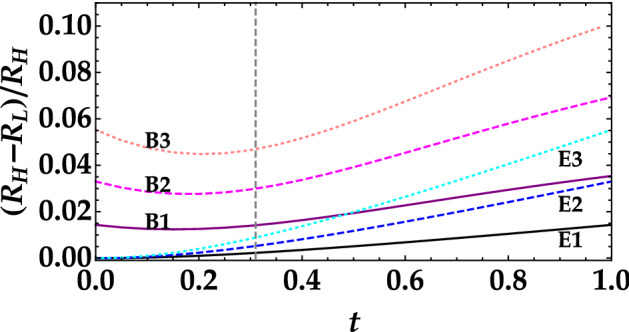
Relative difference between the noise reduction factor measured for high and low mean values of the thermal state as a function of the transmittance efficiency *t*. For all cases $\langle m_{L} \rangle =0.1$. Black and purple solid lines correspond to $\langle m_{H} \rangle =0.2$, blue and magenta dashed lines to $\langle m_{H} \rangle =0.3$, and cyan and pink dotted lines to $\langle m_{H} \rangle =0.4$. Black, blue and cyan lines refer to Eve, while purple, magenta, and pink to Bob. The vertical line corresponds to $t=t^*$, that is the threshold value over which Bob measures $R>1$.

In Fig. 7 we show the theoretical behavior of the noise reduction factor measured by Bob as a function of the transmittance coefficient of the signal subtracted by Eve. We notice that for Bob it is always possible to discriminate the two states (black and red curves) with the difference between them becoming larger at increasing values of *t*. However, it is important to remark that non all values of *t* are fine for a successfull communication since for $t>t^*=0.31$ the values of *R* are larger than 1. For completeness, in Fig. 8 we also show Eve’s counterpart. The main difference is that Eve is not able to properly discriminate the two signals if the transmission coefficient is too small. We compare the results obtained by Bob and Eve in the same graph to better appreciate Bob’s advantage with respect to Eve in the discrimination process. The direct comparison is shown in Fig. 9, where the relative difference between the noise reduction factor in the two cases is evaluated for three different choices of mean values. Note that also in this case the results are shown as a function of the transmittance coefficient of the signal subtracted by Eve over the entire range [0, 1]. However, for a secure communication we must focus on the values of *t* smaller than $t^*$. Moreover, we can clearly notice that in all cases Bob can discriminate the two signal better than Eve and that the larger the difference between the two mean values the easier the discrimination. Keeping this difference small is also useful to decrease the message leakage rate. This is particularly evident in the region of interest, that is for $t < t^*$. For instance, Bob can still discriminate the two signals when the difference is equal to 0.1, while Eve cannot see any difference.

## Conclusions

In this paper, we developed a novel protocol based on mesoscopic TWB states in order to send a binary message encoded in two single-mode thermal states with different mean values. Such thermal states are alternately superimposed to the portion of TWB generated by Alice and sent to Bob. While in the ideal case, namely in the absence of an eavesdropper’s attack, the state actually sent can be easily retrieved from the measurement of the mean value or from the reconstruction of the photon-number distribution, the situation is more complex in the presence of loss and noise in the communication channel. In the considered case, Eve subtracts part of the signal and substitutes it with a thermal noise source having the same mean value as that of the subtracted light. Under this condition, the evaluation of the mean value is useless, while the calculation of the noise reduction factor for detected photons can be used to decode the message, since it gives the possibility to extract information about the signals superimposed to TWB. In the work we have also discussed the minimum length the sequence of thermal state signal with a given mean value should have in order to allow a successful discrimination process. The proof of principle shown in the paper was obtained with HPD detectors, but for the practical implementation of the protocol we intend to use a different class of photon-number-resolving detectors, namely Silicon photomultipliers^27,28^. Indeed, such detectors have a very good dynamic range suitable to employ more populated states and to make the difference between the TWB and the thermal state signals larger. In such a way, we can preserve nonclassicality for larger values of the transmission efficiency with which Eve subtracts part of the signal, and we can make the state discrimination operated by Bob more successful. Moreover, it could be also useful to investigate the possibility to exploit different nonclassicality criteria that could be more sensitive to loss, such as that based on the second-order field photon-number moments^21,22,29^.

## Methods

In order to investigate the feasibility of the protocol, we consider a multi-mode TWB state produced by parametric downconversion in a $\beta$-Barium-Borate crystal pumped by the fourth harmonics of a Nd:YLF laser regeneratively amplified at 500 Hz. Two portions at frequency degeneracy (523 nm) are spatially and spectrally selected, focused into two multi-mode fibers having 600-$\mu$m core diameter and delivered to two hybrid photodetectors. A single-mode thermal state is produced by sending the second harmonics of the laser to a rotating ground glass disk and selecting a single speckle. A half wave-plate followed by a polarizing cube beam splitter placed on the pathway is used to change the energy of the thermal state and switch from one mean value to the other. The thermal field is superimposed to one portion of TWB and detected together with it. The two detector outputs are amplified, synchronously integrated by two boxcar-gated integrators and acquired. The energy of the pump field is changed in steps by means of a half-wave plate followed by a polarizing cube beam splitter. For each mean value of the pump, 100,000 acquisitions are recorded. By exploiting the self-consistent method extensively explained in^30^, each output of the detection chain, expressed in voltages, can be converted in number of detected photons. The strategy allows us to reconstruct the statistics of light and to calculate all the relevant quantities to characterize the optical states.

## Data Availability

The datasets generated during and/or analysed during the current study are available from the corresponding author on reasonable request.
